# Spermatozoa induce transcriptomic alterations in bovine oviductal epithelial cells prior to initial contact

**DOI:** 10.1007/s12079-020-00575-2

**Published:** 2020-09-03

**Authors:** Qurat Ul Ain Reshi, Janeli Viil, James Ord, Freddy Lättekivi, Kasun Godakumara, Mohammad Mehedi Hasan, Monika Nõmm, Kersti Jääger, Agne Velthut-Meikas, Ülle Jaakma, Andres Salumets, Alireza Fazeli

**Affiliations:** 1 Department of Pathophysiology, Institute of Biomedicine and Translational Medicine, University of Tartu, Ravila 14B, 50411 Tartu, Estonia; 2 Institute of Veterinary Medicine and Animal Sciences, Estonian University of Life Sciences, Tartu, Estonia; 3 Competence Centre on Health Technologies, Tartu, Estonia; 4 Department of Chemistry and Biotechnology, School of Science, Tallinn University of Technology, Tallinn, Estonia; 5 Department of Obstetrics and Gynaecology, Institute of Clinical Medicine, University of Tartu, Tartu, Estonia; 6 Institute of Genomics, University of Tartu, Tartu, Estonia; 7 Academic Unit of Reproductive and Developmental Medicine, Department of Oncology and Metabolism, Medical School, University of Sheffield, Sheffield, UK

**Keywords:** Bovine oviductal epithelial cells, Spermatozoa, Contact co-culture, Non-contact co-culture

## Abstract

The capability of spermatozoa to directly influence maternal gene expression is already established. Indeed, some of the changes induced by spermatozoa may have a direct functional importance in the pre-conceptional period. Although the mechanisms underlying these sperm-maternal interactions are not well characterized, it is possible that they could involve ligands that are released from the spermatozoa. This study therefore aimed to test whether physical contact between bovine spermatozoa and bovine oviductal epithelial cells (BOECs) is a prerequisite for spermatozoa-induced gene expression changes. We used two co-culture models: a contact co-culture model in which spermatozoa interact directly with BOECs, and a non-contact co-culture model in which an insert with the pore size of 0.4 μm was placed between spermatozoa and BOECs. Messenger RNA sequencing analysis of BOECs by RNA-seq revealed ten differentially expressed genes in contact system and 108 differentially expressed genes in the non-contact system after 10 h of co-culture. Retinol metabolism pathway and ovarian steroidogenesis pathway were significantly enriched in the non-contact co-culture system. Q-PCR analysis revealed that transcriptional responses can be rapid, with increased expression of four genes (*DHRS3*, *CYP1B1*, *PTGS2*, and *ATF3*) detectable within just 90 min of co-incubation, but with expression levels highly dependent on the type of co-culture system. The findings from our study demonstrate that direct contact with spermatozoa is not necessary to induce changes in gene expression of oviductal epithelial cells, suggesting that spermatozoa may be able to signal to maternal tissues in advance of their arrival.

**Electronic supplementary material:**

The online version of this article (10.1007/s12079-020-00575-2) contains supplementary material, which is available to authorized users.

## Background

The oviduct, also called fallopian tube in mammals, is an important site where several crucial cellular and molecular events have to occur between the cells of the female reproductive tract and gametes of both sexes to have a successful conception. The reproductive tract communicates and interacts with spermatozoa during the pre-conception period in diverse ways in order to facilitate their maturation, transportation, and survival, and establish proper physiological conditions for fertilization (Fazeli et al. 2004; Holt and Fazeli 2016; “The fallopian tubes,” 1989). Spermatozoa interact with oviductal cells at two levels - physical contacts and molecular interactions. Physical contacts include sperm cells swimming through the female reproductive tract aided by the flow of the oviductal fluid, the ciliary beating of the epithelial cells, and contractions of the female reproductive tract. On the molecular level, spermatozoa interact with oviductal cells through their surface proteins that have been shown to alter the gene expression of the epithelial cells in the oviduct (Alvarez-Rodriguez et al. 2019; Fazeli et al. 2004; López-Úbeda et al. 2015). Oviduct cells produce a diverse range of secreted proteins, extracellular vesicles (EV) and other specific signaling molecules, some of which are implicated in sperm-oviduct interactions (Almiñana and Bauersachs 2019; Jamaludin et al. 2019). However, less is known about the signaling molecules released from spermatozoa that affect epithelial cells of the female reproductive tract.

Several in vivo studies have reported that oviduct responds to the arrival of spermatozoa through changes in its transcriptome as well as in other morphological processes including ciliary beating and secretion of bioactive compounds which mediate transport of the sperm cells (Almiñana and Bauersachs 2019; Fazeli et al. 2004; Kodithuwakku et al. 2007). In a study conducted on female turkeys, it was found that 1% of the genes in the sperm storage tubules were differentially expressed 48 h after sperm insemination suggesting that sperm caused alterations in the gene expression of the female reproductive tract (Long et al. 2003). Similar studies in pigs have shown that several genes in sow’s oviductal cells are differentially expressed between naturally inseminated and non-inseminated animals (López-Úbeda et al. 2015). Another piece of evidence supporting the idea of spermatozoa affecting the gene expression of oviductal cells came from a study where authors used mutated mice (T145H mutation) with spermatogenic arrest, which are only able to produce seminal plasma but not spermatozoa. When such infertile male mice were mated with normal female mice, no changes in expression levels were detected in adrenomedullin and prostaglandin-endoperoxidase synthase 2 (*PTGS2*) genes but after mating with normal male mice both of these genes were up-regulated suggesting that sperm cells indeed alter the expression of specific genes in oviductal epithelium (Fazeli et al. 2004).

Studies suggest that the entry of spermatozoa to the reproductive tract elicits a uterine inflammatory response and therefore alters the innate as well as the acquired local immune system (Rodríguez-Martínez et al. 2009; Rozeboom et al. 1998). Natural or artificial insemination in the cervix or upper reproductive tract in porcine females has been shown to modify, mostly down-regulate, the expression of various genes responsible for modulating the local immune response, including chemokine and interferon-gamma signaling genes, and JAK/STAT pathway-related genes (Alvarez-Rodriguez et al. 2019). Furthermore, after mating the gene expression of cytokine related genes such as *TNFSF11* (TNF super-family member 11) and *ADGRB2* (Adhesion G Protein-Coupled Receptor B2) was also down-regulated (Alvarez-Rodriguez et al. 2019), a response usually elicited to eliminate invading microorganisms. Therefore, the mechanisms of sperm tolerance and survival appear to be counteracting such inflammatory responses, although their extensive details are yet to be discovered.

Although it has been established that spermatozoa induce changes in the gene expression of the oviductal epithelium, it has remained mostly unknown by which mechanisms spermatozoa communicate/interact with epithelial cells to elicit such responses. It is also not fully known what type of molecular receptors or contact points between spermatozoa and reproductive tract epithelium are necessary. Among various communicating agents and messenger molecules, EVs that contain a mixture of proteins, peptides, miRNAs, lipids as well as DNA fragments are suggested to play an important role in this inter-cell communication (Almiñana and Bauersachs 2019; Ferraz et al. 2019). Several studies have shown that seminal plasma contains diverse types of EVs such as prostasomes, small vesicles secreted by prostatic epithelial cells, which are important for sperm hyper-motility and acrosome reaction (Aalberts et al. 2013). Prostasomes bind to spermatozoa in the uterus and are carried into the oviduct in association with spermatozoa (Aalberts et al. 2013), however, it is equally likely that prostasomes may be involved in sperm-oviduct dialogue, as well. It is still unknown if spermatozoa themselves release EVs, which could mediate communication with oviductal cells by modulating their gene expression.

The current study was set out to investigate whether mature spermatozoa release biomolecules that could trigger gene expression changes in oviductal cells by using contact and non-contact co-culture systems with bovine spermatozoa and primary bovine oviductal epithelial cells (BOECs). For this study we used a novel non-contact co-culture system to analyse whether the spermatozoa could communicate with oviductal epithelial cells, when not in direct contact with them.

## Materials and methods

### Isolation and culture of primary BOECs

Oviducts with attached ovaries were collected from the slaughterhouse and transported to the laboratory in saline at 37 °C within 4 h after animal slaughter. Only the oviducts of the early stage of the estrous cycle (bright red *corpus luteum*, 0–4 days after ovulation) were selected for isolation (Ireland et al. 1980). The ovary and the connective tissue were removed from the oviducts with a scalpel, followed by washing with wash buffer-1 (DPBS supplemented with Amphotericin B (1 μl/ml) and 1X Penicillin/Streptomycin (10 μl/ml)). The ampulla and the isthmus part of the oviducts were separated by cutting the oviducts at the ampullary-isthmic junction and were handled separately. The mucosa was extracted by squeezing the oviduct gently with a sterile glass slide and transferred into a tube containing wash buffer-1. The cells were washed thrice, two times in wash buffer-1 and once in wash buffer-2 (DPBS supplemented with Amphotericin B (1 μl/ml), Penicillin/Streptomycin (10 μl/ml) and 5% FBS) and between each wash, the cells were allowed to settle down after which the supernatant was removed. The final pellet of the cells was resuspended in culture media (DMEM/F12 supplemented with 10% FBS, Amphotericin B (1 μl/ml) and Penicillin/Streptomycin (10 μl/ml)), transferred to T-25 flasks and incubated in a humidified atmosphere with 5% CO_2_ at 38.8 °C. The cells were allowed to attach for the next 3 days without changing the media. Afterwards, the cells were trypsinized and transferred to a T-75 flask, and media was changed every 48 h until confluency of 80% was attained. The cells had been split four times before they were co-cultured with spermatozoa.

### Immunofluorescence analysis of bovine epithelial cells (BOECs)

BOECs were grown on coverslips and then fixed with 4% paraformaldehyde for 15 min at room temperature, and in order to permeabilize the cell membrane, cells were treated with cold methanol for 10 min on ice. Thereafter, 4% normal goat serum was used for blocking for 1 h at room temperature. The cells were then incubated with a mix of anti-Cytokeratin (C2562, 1:250, Sigma-Aldrich) and anti-Vimentin (PLA0199, 1:250, Sigma-Aldrich, USA) primary antibodies in blocking buffer overnight at 4 °C. Negative control was incubated with a blocking buffer lacking any of the primary antibodies. Secondary antibody incubation was done using goat anti-mouse (conjugated to Alexa Fluor 488,1:500) and goat anti-rabbit (conjugated to Alexa Fluor 594, 1:500) secondary antibodies (both Invitrogen, Thermo Fisher Scientific, Eugene, USA) in blocking buffer for 1 h in the dark at room temperature. After incubation, the nuclei were stained using Hoechst 33342 (1:2000, Thermo Fisher Scientific) for 3 min and then the coverslips were mounted with Fluorescence Mounting Medium (Dako, Denmark). Images were taken with a Leica DM5500B microscope equipped with Leica DFC310 camera (Leica, Wetzlar, Germany) and processed with ImageJ (Schindelin et al. 2012).

### Spermatozoa washing and culture

Within each of the two experiments (RNAseq and qPCR), we used multiple semen straws all deriving from the same ejaculate from the same bull. For each day that the experiment was carried out, nine straws of frozen bovine semen were thawed in 37 °C sterile water bath for 30 s. Contents of the three straws (250 μl of semen in each straw) were pooled and deposited onto 4 ml of 60% isoosmotic Percoll® solution (GE Healthcare, 17–0891-02, Sweden) prepared as described previously (Aldarmahi et al. 2014), with slight modifications. The modifications included making 100% Percoll by mixing 1X HEPES with Percoll in 1:9 ratio, while 60% Percoll was prepared by diluting 100% Percoll with supplemented Sperm-TALP media. Percoll with semen was centrifuged for 20 min at 300×g at room temperature and the pellet was washed with pre-warmed EV-depleted Sperm-TALP (NaCl: 0.005 g/ml, KCl: 0.23 mg/ml, NaH_2_CO_3_: 0.002 g/ml, lactic acid 60% /ml, NaH_2_PO_4_: 0.034 mg/ml, CaCl_2_: 0.308 mg/ml, MgCl_2_: 100 mM 15 ul/ml, HEPES: 0.0023 g/ml, Gentamycin 0.5ul/ml, sodium pyruvate: 0.1ul/ml, and BSA: 0.6 mg/ml) media and centrifuged again for 5 min at 400×g. Due to the presence of BSA (AppliChem, A1391, 0050, Germany) in the media, EV depletion was performed in order to minimize the number of BSA-derived particles in the media by filtering the BSA solution through 100 kDa Amicon® Ultra-15 Centrifugal Filter Unit (R9CA01172, Ireland) (Kornilov et al. 2018). The final pellet was resuspended in 1 ml of pre-warmed EV-depleted Sperm-TALP media and the concentration of washed spermatozoa was determined. The concentration was adjusted to 1 × 10^6^/ml and the motility was analysed in five different fields under the microscope with 40X magnification. Spermatozoa were processed on three different days for RNA sequencing experiment and the measured average motility post-thawing was 60% (day-1), 65% (day-2) and 60% (day-3). Similarly for experiments with q-PCR, the average motility of spermatozoa on three different days was 65% (day-1), 70% (day-2) and 60% (day-3) post-thawing. All procedures were performed under aseptic conditions and the washed spermatozoa were immediately used for co-culturing with BOECs.

### Co-culturing BOECs and spermatozoa in a contact and non-contact co-culture model

Frozen BOECs from isthmus and ampulla were thawed and first cultured separately. Afterwards, the cells from ampulla and isthmus were mixed and plated onto a 12-well plate until they attained 80% confluency, at which point cells were divided across experimental replicates. Before the addition of spermatozoa, the BOECs were washed once with pure DMEM/F12 media, followed by washing with EV depleted sperm-TALP media.

**Fig. 1:**
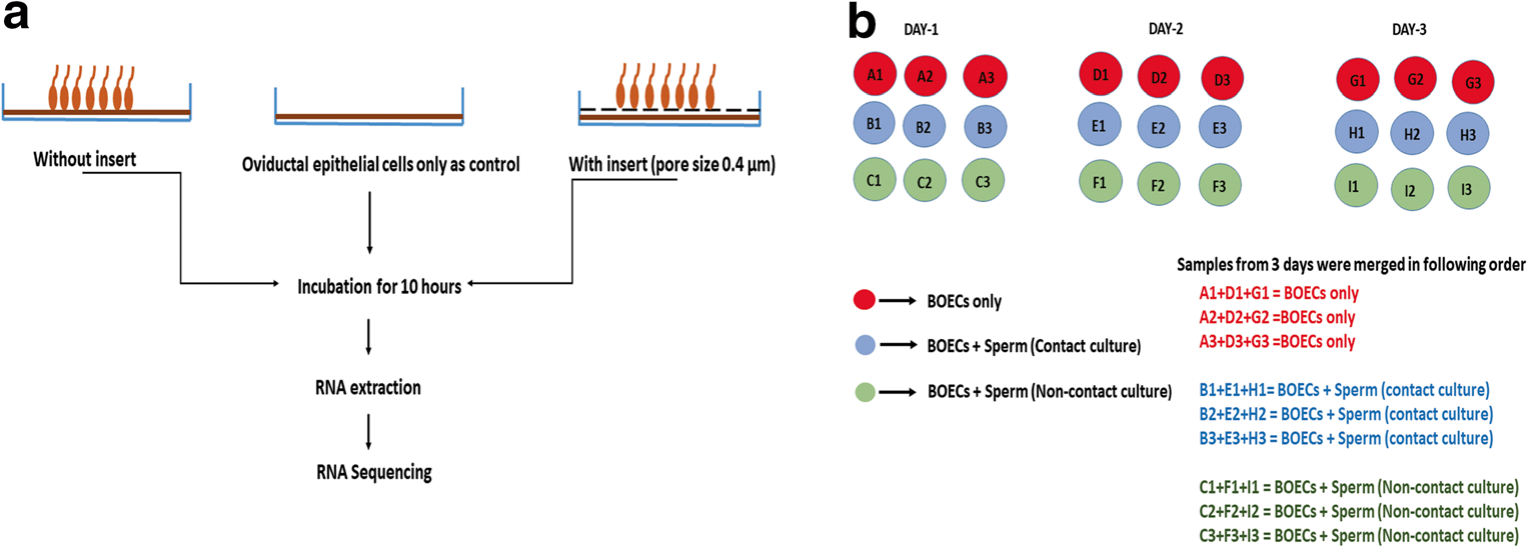
**a** Schematic illustration of the two co-culture models used in the experimental set up where in one model the spermatozoa interact directly with the BOECs monolayer (contact co-culture) and in other model an insert with a pore size of 0.4 μm was used to separate BOECs from spermatozoa (non-contact co-culture). BOEC monolayer with no spermatozoa served as the control for the experiment. **b** Schematic diagram representing the number of replicates for each group and the procedure for merging the samples for RNA sequencing, RNA obtained was mixed across the three days to pool the variation arising from different days

In order to identify the genes that were differently expressed in BOECs in response to spermatozoa, we used two co-culture models and an independent BOEC culture as the control group. In the first co-culture model, the spermatozoa were allowed to directly interact with BOECs - hereinafter referred to as contact co-culture. In the second co-culture model, an insert with a pore size of 0.4 μm (Thincert cell culture insert, Greiner Bio-One GmbH, Kremsmünster, Austria) was used to spatially separate the spermatozoa and BOECs - hereinafter referred to as non-contact co-culture. Equal amounts of washed bovine spermatozoa (1 × 10^6^ spermatozoa/ml) were added to BOECs in both co-culture models and co-incubation was performed for 10 h, after which RNA was extracted from the BOECs for sequencing. The control group BOECs were processed equivalently. The aforementioned experiment was repeated three times on three different days, using a different aliquot of the same primary cells. The source of spermatozoa was also kept constant (all thawed semen straws derived from the same ejaculate). In each repetition of the experiment, both co-culture models and the control group were cultured across three replicate wells. After the three repetitions of the experiment, the RNA from the wells was pooled across all three repetitions of the experiment, so that a final experimental replicate consisted of three pooled wells, each of which originated from a different repetition of the experiment. The pooling of wells across the experimental repetitions was conducted in order to alleviate batch variation as it was not possible to perform the whole experiment with all replicates in a single day. The experimental design is explained in more detail in Fig. 1.

In order to unfold the dynamics of gene expression change for the selected genes of interest, additional experiments were performed using the same co-culturing conditions with varying incubation periods during which the spermatozoa were allowed to exert an effect on the BOECs. The same source of BOECs as in the RNA sequencing experiment was used. The spermatozoa, however, originated from a different ejaculate of the same bull. The experiment was repeated for four different co-incubation periods for both the co-culture models and the control BOECs. The time periods were 30 min, 90 min, 2.5 h, and 4 h. The experiments were conducted in three identical repetitions on three different days resulting in three replicates for each experimental group.

### RNA extraction, sequencing library preparation, and RNA sequencing

After the respective co-incubation periods, the media was discarded, followed by RNA extraction using QIAzol® Reagent (Qiagen, 79,306, USA) and isopropanol precipitation method, according to the manufacturer’s protocol. Briefly, 500 μl of guanidinium thiocyanate (QIAzol® reagent) was added to BOECs in 12-well plate and the cells were left at room temperature for 10 min. The contents were thoroughly homologized with a pipette, transferred to a clean tube and 100 μl of chloroform was added. Once the samples were vortexed for 15 s, they were immediately centrifuged at 12,000×g for 15 min at 4 °C for phase separation. The aqueous phase containing RNA was transferred to a new tube and RNA was precipitated by adding 250 μl of isopropanol to the aqueous phase and incubating samples at room temperature for 20 min. Samples were centrifuged for 30 min at 16,000×g at 4 °C and then the RNA pellet was washed thrice in 500 μl of 75% ethanol. The final RNA pellet was air dried, resuspended in 30 μl of nuclease free water and heated at 60 °C for 10 min.

The extracted RNA was quantified using Qubit™ RNA HS Assay Kit according to the manufacturer’s protocol (Q32852, ThermoFisher Scientific). The quality of the extracted RNA was determined with the Bioanalyzer Automated Electrophoresis instrument (Agilent technologies, Santa Clara, CA) and Agilent RNA 6000 nano Kit (Agilent technologies).

Smart-seq2 methodology (Picelli et al. 2014) with slight modifications was employed to generate RNA sequencing libraries. Instead of single cells, we used 20 ng of total RNA for cDNA synthesis and 10 cycles of PCR for pre-amplification. KAPA HiFi DNA polymerasewas replaced with Phusion High-Fidelity DNA Polymerase (Thermo Scientific) which is compatible with the original protocol. 2 μl of diluted cDNA was applied to dual-index library preparation using Illumina Nextera XT DNA Sample Preparation Kit (FC-131-1024). Ampure XP beads (Beckman Coulter) were used for all clean-up steps and for size selection of 200–700 bp. All samples were pooled into a single library by equal concentration and sequenced on Illumina NextSeq500 using High Output Flow Cell v2.5 (single-end, 75 bp).

### Read processing, alignment, and counting for RNA-seq data analysis

Read quality was assessed with FASTQC (Brown et al. 2017), trimming of reads for adapter sequences and the removal of low-quality reads was done using trimmomatic (Bolger et al. 2014). Reads were aligned to the *B. taurus* genome assembly (ARS-UCD1.2). Hisat2 was used for read alignment to the reference genome (Kim et al. 2019). Read alignment was performed with default parameters and with the inclusion of splice site information derived from the corresponding Ensembl *B. taurus* annotation file (ARS-UCD version 1.2.97). Gene-level read counts were obtained using featureCounts (Liao et al. 2014) with default parameters, using the Ensembl *B. taurus* annotation file (version 1.2.97) for the ARS-UCD1.2 genome assembly to obtain feature information.

### Differential expression analysis

Differential expression analysis of gene-level counts was carried out in R version 3.6.1 using the edgeR package (Robinson et al. 2010). The genes were filtered to exclude genes that were assigned less than 10 reads for all the samples in one experimental group. The genes remaining in the analysis were subjected to differential expression testing. Tagwise dispersion estimates were obtained based on the trended dispersions, and statistical comparisons were performed using a generalised linear model followed by likelihood ratio tests. The *P* value was adjusted for multiple testing to obtain the false discovery rate (FDR) value using the default Benjamini-Hochberg method (Benjamini and Hochberg 1995). The results were considered statistically significant at false discovery rate (FDR) ≤ 0.05.

### Pathway enrichment analysis

Pathway over-representation analysis was carried out using R-package clusterProfiler (Yu et al. 2012) and KEGG Pathway database annotations. Pathway analyses were carried out separately for up- and down-regulated genes which were differentially expressed at FDR ≤ 0.05.

### Quantitative reverse transcription-PCR

cDNA was synthesized using a mixture of oligo(dT) and random primers (FIREScript RT cDNA synthesis kit, Solis BioDyne, Tartu, Estonia). q-PCR was performed in triplicates for each biological sample and the cDNA products were amplified using the EvaGreen assay system (Solis BioDyne, Tartu, Estonia). The following program was used for amplification: 95 °C for 15 min, followed by 40 cycles of 95 °C for 20 s, 62 °C for 20 s and 72 °C for 20 s. A dissociation curve was performed to verify the purity of the PCR product. q-PCR data was analysed using a comparative CT method and the relative expression of RNA was calculated based on the 2^-ΔΔ*CT*^*method* (Winer et al. 1999). Beta-2-microglobulin (*B2M*) and TATA-binding protein (*TBP*) genes were used for normalisation. Primers were designed using Primer-BLAST and the preference was given to the sequences that spanned exon-exon junction. Primer sequences for all genes can be found in Table 1.

**Table 1:** Primer list for q-PCR

Gene name	Primer sequence (5′-3′)
*PTGS2*	Forward: TGAGGAACTTACAGGAGAGAAGGA
	Reverse: TCTACCAGAAGGGCGGGATA
*CYP1B1*	Forward: GGCTGACTCTGGCGATGGT
	Reverse: CTGCACTTCCGAATACCTGGTG
*DHRS3*	Forward: TATTTCCGGGATGGTCTGTGC
	Reverse: TATATTCCTGCCGTTCAACCAGT
*ATF3*	Forward: AGTGGATACAGGAGCAAAATGATG
	Reverse: CAGAGGCACTGACTTCCGAG
Beta-2-microglobulin	Forward: CTGCAAGGATGGCTCGCTT
	Reverse: GAATCTTTGGAGGACGCTGGA
TATA binding protein	Forward: GCACAGGAGCCAAGAGTGAA
	Reverse: TCCCCACCATGTTCTGAATCTT

### Statistical analysis and visualization

Statistical analyses of qPCR-derived gene expression results were carried out in R version 3.6.1. For each gene of interest, a linear mixed model was fitted using the lmer function from the lme4 package (Bates et al. 2015). For each model, treatment and time were included as interacting fixed effect terms and experimental batch was included as a random effect to account for the collection of samples from three experimental batches. Inter-group comparisons at specific timepoints were performed as post-hoc pairwise comparisons of estimated marginal means with Tukey correction for multiple testing, for which the emmeans function from the emmeans package was used. All graphs were generated using ggplot2 version 3.2.0 (Wickham 2009), except for the Venn diagram, for which the GOplot package version 1.02 was used (Walter et al. 2015).

## Results

### Immunocytochemical localization of epithelial markers in BOECs

**Fig. 2:**
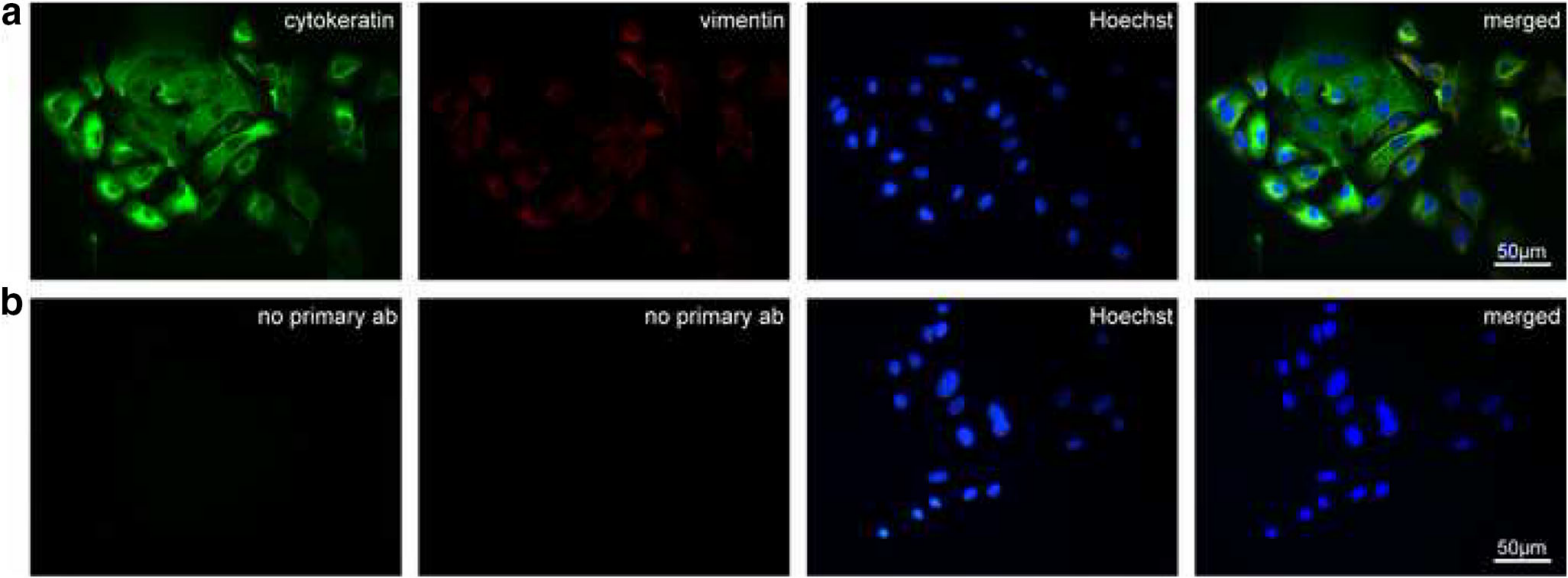
Cytokeratin and Vimentin expression in primary bovine oviductal epithelial cells. **a** Cells displaying strong signal after staining with anti-Cytokeratin antibody (green) and faded signal with anti-Vimentin antibody (red). Nuclei were stained with Hoechst (blue). **b** Negative control staining without primary antibodies. Nuclei were stained with Hoechst

Cytokeratins are important and major intermediate filaments of epithelial cells while Vimentin is the intermediate filament expressed in fibroblasts (Zeisberg and Neilson 2009). We used antibodies against cytokeratin and vimentin to evaluate if the primary cell culture we had established consisted mostly of epithelial cells, without fibroblast contamination. Incubation of the isolated cells with anti-Cytokeratin antibody showed a strong positive signal (Fig. 2a), whereas staining with anti-Vimentin antibody produced a fade signal which confirmed the presence of epithelial cells mostly.

### RNA-seq results and differential gene expression

Sequencing of the mRNA libraries yielded 6.2 ± 1.2 million reads (mean ± SD) per sample. After filtering for read quality, 98.7 ± 0.2% of the reads remained, out of which 96.3 ± 0.2% aligned to the *B. taurus* genome assembly (ARS-UCD1.2) at least once. Read counts were summarized at the gene level, and after filtering to remove genes considered not to be expressed in any of the groups, 10,636 genes remained and were subsequently tested for differential expression.

**Fig. 3:**
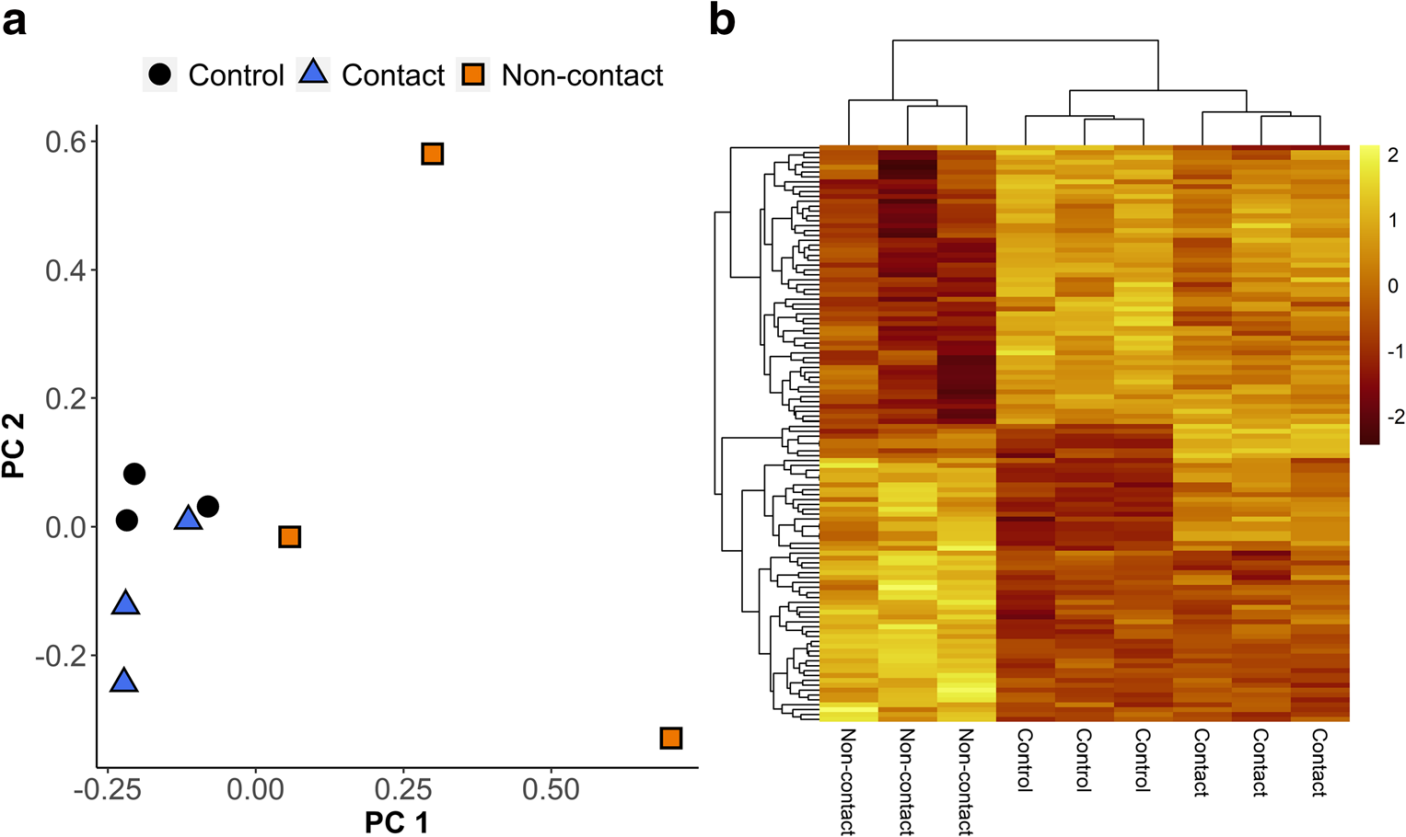
**a** Principal Component Analysis (PCA) plot of inter-sample distances calculated based on log2 fold changes in the top 500 most variably expressed genes in BOECs under control conditions (black), contact co-culture with spermatozoa (blue) and non-contact co-culture with spermatozoa (orange) after 10 h of incubation. **b** Heatmap of standardised (z-score) CPM values of genes that were differentially expressed either in response to contact or non-contact co-culture with spermatozoa, with lighter shade denoting higher levels of relative expression and darker shades denoting lower levels of relative expression

**Fig. 4:**
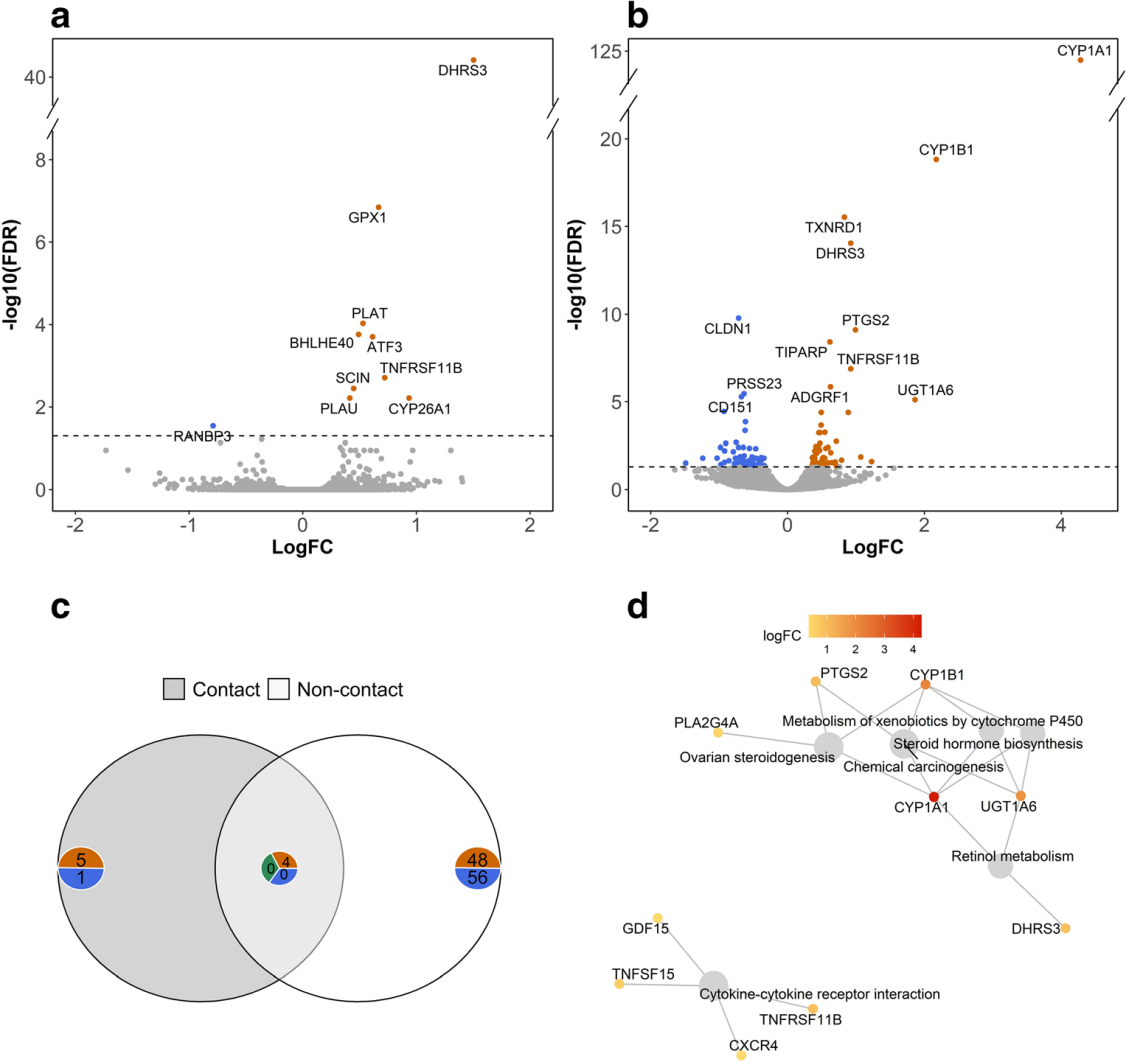
**a** Volcano plot of the differentially expressed genes in BOECs in the contact co-culture model; the genes that were found to be statistically significant (FDR ≤ 0.05) are highlighted. **b** Volcano plot of the differentially expressed genes in BOECs in the non-contact co-culture model; the genes that were found to be statistically significant (FDR ≤ 0.05) are highlighted. **c** The number of statistically significant (FDR ≤ 0.05) differentially expressed genes detected exclusively in either of the co-culture models or common to both of the model systems. The number of genes is presented on blue or orange backgrounds for downregulated or upregulated genes, respectively. The number of contra-regulated genes is presented on the greed background. **d** Network plot based on the pathways detected to be significantly enriched among the upregulated genes in BOECs in the non-contact co-culture model and the genes which contributed to the enrichment of these pathways

Non-contact co-culture model samples completely segregated from both control and contact co-culture model samples on the first principal component axis but exhibited considerably more noticeable intra-group variation compared to the other two groups (Fig. 3a). Contact co-culture with spermatozoa induced only minor changes to gene expression profiles of BOECs, with nine genes upregulated and one gene down-regulated (Fig. 3b). Complete list of differentially expressed (DE) genes in contact co-culture can be found in Supplementary Data (Table SI). The most upregulated gene in response to contact co-culture was *DHRS3*, while the only downregulated gene was *RANBP3* (Fig. 4a). Surprisingly, the non-contact co-culture treatment induced more extensive gene expression changes in BOECs (52 genes upregulated, and 56 genes downregulated; Fig. 3b). List of DE genes in non-contact co-culture can be found in supplementary data (Table SII). The topmost upregulated genes in response to non-contact co-culture were metabolic enzyme genes, including the mono-oxidases *CYP1A1* and *CYP1B1,* as well as *TXNRD1, DHRS3,* and *PTGS2* (Fig. 4b)*.*

Four upregulated genes (*PLAU, TNFRSF11B, SCIN,* and *DHRS3*) were common to both contact and non-contact co-culture (Fig. 4c). Of the four common upregulated genes, DHRS3 exhibited a higher degree of upregulation in contact co-culture (log_2_FC = 1.5) compared to non-contact co-culture (log_2_FC = 0.92). The other six DE genes in response to contact co-culture were unique to this treatment and interestingly included *ATF3*, a known negative regulator of *PTGS2* (Hellmann et al. 2015).

### Pathway over-representation analysis (non-contact co-culture)

To identify pathways that were over-represented among genes that were differentially expressed in BOECs in response to non-contact co-culture with spermatozoa, pathway over-representation analyses was conducted separately for significantly up-regulated and down-regulated genes based on KEGG pathway annotations for *B. taurus*.

Among up-regulated genes, six pathways were significantly enriched at FDR < 0.05, of which the most pertinent were retinol metabolism (bta00830), steroid hormone biosynthesis (bta00140), ovarian steroidogenesis (bta04913), and cytokine-cytokine receptor interaction (bta04060) (Table 2). The topmost enriched pathways also featured some of the most strongly upregulated genes: five featured *CYP1A1*, four featured *CYP1B1*, and two featured *PTGS2* (Fig. 4d). No significantly enriched pathways were detected among down-regulated genes.

**Table 2:** Top six enriched KEGG pathways detected among 52 significantly upregulated genes (FDR <0.05) in BOECs in response to non-contact co-culture with spermatozoa following 10 h of incubation. Columns ‘Gene IDs’ and ‘Gene count’ show the significantly upregulated genes and the number of significantly upregulated genes that belong to the corresponding pathway

Pathway ID	Pathway description	FDR	Gene IDs	Gene count
bta04913	Ovarian steroidogenesis	0.003	CYP1A1, CYP1B1, PTGS2, PLA2G4A	4
bta00830	Retinol metabolism	0.012	CYP1A1, DHRS3, UGT1A6	3
bta05204	Chemical carcinogenesis	0.012	CYP1A1, CYP1B1, PTGS2, UGT1A6	4
bta00140	Steroid hormone biosynthesis	0.013	CYP1A1, CYP1B1, UGT1A6	3
bta00980	Metabolism of xenobiotics by cytochrome P450	0.039	CYP1A1, CYP1B1, UGT1A6	3
bta04060	Cytokine-cytokine receptor interaction	0.039	TNFRSF11B, GDF15, TNFSF15, CXCR4	4

### Short-term expression patterns of genes affected by contact and non-contact co-culture (qPCR)

To examine how rapidly the BOECs respond to the stimuli of spermatozoa by changing the gene expression, we performed q-PCR analysis of four genes of interest for both contact and non-contact co-culture models after 30, 90, 150, and 240 min of co-culture. Specifically, we examined the expression of one gene shown to be up-regulated by RNA-seq in both treatments (*DHRS3*), two up-regulated genes only in response to non-contact co-culture (*CYP1B1* and *PTGS2*), and one up-regulated gene only in response to contact co-culture (*ATF3*).

**Fig. 5:**
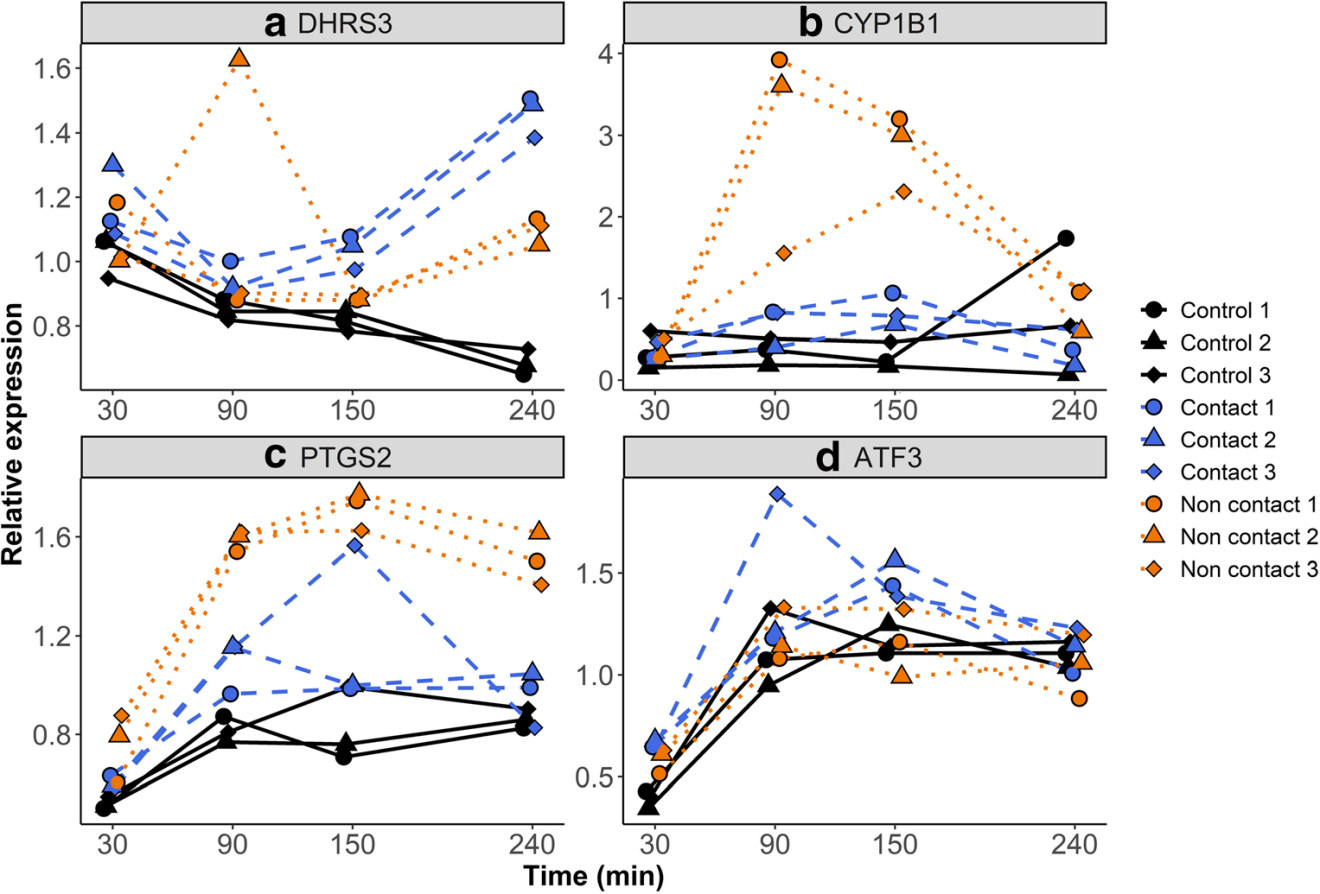
Quantitative real-time PCR-derived relative expression values of four genes in BOECs under control conditions (black, solid lines) and in response to contact co-culture with spermatozoa (blue, dashed lines) or non-contact co-culture with spermatozoa (orange, dotted lines). Samples were collected after 30, 90, 150, and 240 min of incubation. Each point represents an individual primary cell culture, with point shapes representing the experimental batch from which the sample was derived (circles: batch 1, triangles: batch 2, diamonds: batch 3). Relative expression values were calculated using the 2^-ΔΔ-CT^ method

*DHRS3* showed increased expression in response to both contact and non-contact co-culture (Fig. 5a), but was stronger in response to contact, in which a statistically significant increase in expression was detectable starting from 30 min (*P* = 0.02) and a highly prominent increase observed at 240 min. In contrast, a significant increase in *DHRS3* in response to non-contact culture was not observed until 240 min had elapsed. *DHRS3* expression was analysed following the removal of a prominent outlier in the non-contact group (2nd replicate, 90 min).

Interestingly, the expression of *CYP1B1* was prominently increased in response to non-contact after 90 min (*P* < 0.001), but the expression returned to the level of control samples at 240 min (Fig. 5b). Similarly *DHRS3, CYP1B1* expression showed a weaker increase in response to contact co-culture which peaked at 150 min (*P* = 0.04) and subsided afterwards.

*PTGS2* expression was similarly increased at 30 min in response to non-contact (*P* = 0.003), but conversely showing a sustained increase in expression levels of *PTGS2* until the final timepoint. In contact co-culture, *PTGS2* showed a modest increase in response to spermatozoa observable at 90 min (P = 0.02), but this increase in the expression levels of the gene was not sustained until the final timepoint (Fig. 5c).

*ATF3* expression (Fig. 5d) was increased at 30 min in response to both contact (P < 0.001) and non-contact co-culture (P < 0.001). However, the expression of *ATF3* was not increased at any other subsequent timepoint in non-contact co-culture but remained significantly increased in response to contact co-culture until and including 150 min timepoint (P = 0.04) before subsiding.

## Discussion

**Fig. 6:**
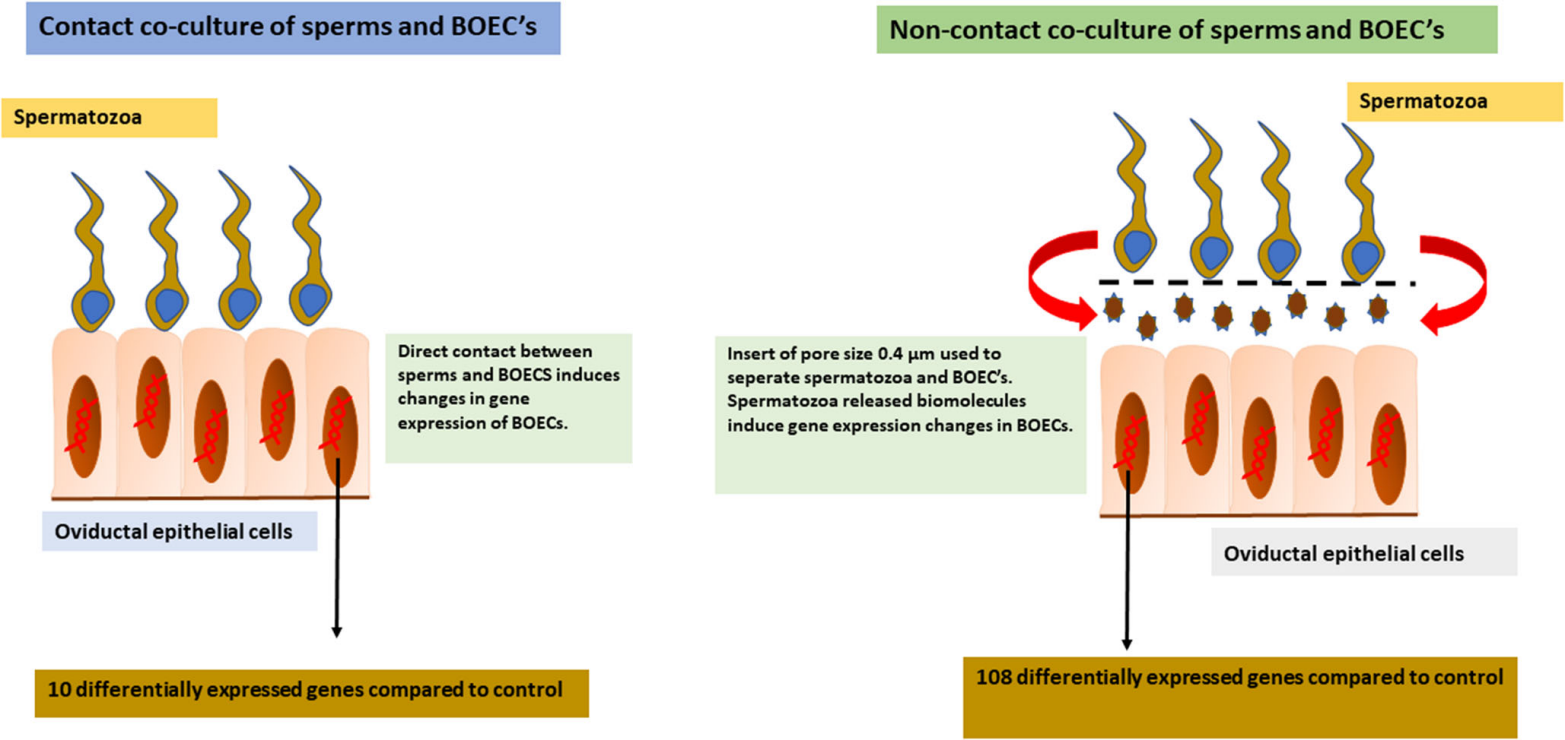
Overall layout and findings of the study, where spermatozoa are capable of inducing gene expression changes in oviductal epithelial cells but the gene expression levels are highly dependent on the type of co-culture system

While the capacity of spermatozoa to directly exert changes in maternal physiology has been known for some time (Fazeli 2011; Fazeli et al. 2004; Kodithuwakku et al. 2007; López-Úbeda et al. 2015) the possible mechanisms of communication between spermatozoa and maternal reproductive tract have been relatively under-explored. In this study, we tested whether spermatozoa are capable of communicating remotely with maternal cells by characterising the transcriptomic responses in BOECs incubated either in direct contact with spermatozoa or in a non-contact co-culture system as in general illustrated in Fig. 6.

Remarkably, not only were spermatozoa capable of inducing alterations to BOECs gene expression in the absence of direct physical contact, but the extent of gene expression changes was substantially greater than that induced by direct contact with spermatozoa (108 DEGs in response to non-contact co-culture compared to only 10 DEGs in response to contact co-culture). This implies the existence of molecular signals released by the spermatozoa which are capable of evoking functional responses in the maternal tract. Nonetheless, we also found a small number of genes (e.g. *ATF3*) that were differentially expressed only in response to contact co-culture. Thus, it can be inferred that some responses require direct contact between spermatozoa and oviductal cells.

One hypothesis to explain the greater magnitude of differentially expressed genes in BOECs in non-contact compared to contact co-culture systems after 10 h of incubation is that the responses induced by direct contact may be more immediate than responses induced by non-contact. Conveyed by mediators of intercellular communication, the signals in non-contact system would take more time to reach the target cells, and therefore may subside after a longer time period even if the initial responses are of equal magnitude. Indeed, the expression levels of *CYP1B1* and *PTGS2*, which were not affected by contact co-culture based on RNAseq data, were nonetheless increased in response to contact co-culture during the 4-h observation period of the qPCR experiment. However, the expression levels of both of these genes were considerably lower compared to the upregulation induced by non-contact culture which was highly pronounced even within this short timeframe. Assuming that the signals presented to BOECs through the non-contact system are also present in contact co-culture, both RNAseq and qPCR results suggest that signals released from the spermatozoa surface have strong influences on BOECs gene expression. However, the observed smaller number of differentially expressed genes in the contact co-culture system also suggests that many responses could be suppressed by ligands present only on the sperm surface. Indeed, *ATF3*, which was upregulated in response to contact but not non-contact culture, has been known to have negative regulatory effects on the expression of *PTGS2* during acute inflammation (Hellmann et al. 2015, p. 3). Inhibitory effects of *ATF3* may therefore partly explain why *PTGS2* upregulation was considerably weaker in response to contact culture compared to non-contact culture, as shown by the results of the time series experiment.

Results of the time series experiment revealed two genes involved in ovarian steroidogenesis pathway- *PTGS2* and *CYP1B1* - to be upregulated in both co-culture models within a short time frame. Prior studies have also shown that the gene expression of *PTGS2* is upregulated in bovine oviductal epithelial cells in response to spermatozoa in a contact culture. It is also suggested that *PTGS2* enhances the oviductal ciliary motility and accelerates the transport of spermatozoa towards the oocyte (Kodithuwakku et al. 2007).

Regarding the putative functions of the responses to non-contact co-culture, pathway enrichment analysis revealed several pathways that were enriched with upregulated genes, the most enriched of which was the retinol metabolism pathway. Proper retinoic acid signalling is crucial for normal early embryo patterning and development. One of the key players in this pathway is *DHRS3*, which attenuates the synthesis of retinoic acid signalling (Kam et al. 2013, p. 3). Resulting from the time series experiment, we observed *DHRS3* to be significantly upregulated in both co-culture systems within a short timeframe (Fig. 4a). Ovarian steroidogenesis was also revealed as one of the significantly enriched pathways, which is pertinent as there is evidence that the steroid environment ensures successful oocyte fertilization (Yoshimura et al. 1986). The potential of the oviduct to secrete steroids has been demonstrated in a study conducted in porcine and has been suggested to have role in controlling early periods of pregnancy (Martyniak et al. 2018). Fertilization and early embryonic development are supported by secretion of oviductal factors, the regulation of which is controlled by the action of oviductal steriods (Ballester et al. 2014; Li and Winuthayanon 2017; Wollenhaupt et al. 1999). Ovarian steroids play a significant role in the synthesis of cytoplasmic factors that induce normal sperm head decondensation and formation of the male pronucleus (C 1977; Thibault et al. 1975). Defective sperm head decondensation has been associated as one of the factors responsible for the failed fertilization (Esterhuizen et al. 2002). The relevance of these pathways enriched in response to spermatozoa remains elusive, however the collective notion suggests that these pathways might have some role to play in terms of embryo development and fertilization. We assume that the count of spermatozoa being in millions is because not all the spermatozoa succeed in reaching the point of fertilisation, instead some of them die and some induce changes in the female reproductive tract and periconception milieu which are important with respect to successful fertilization.

Although our results indicate that some messenger biomolecules are released from spermatozoa, which in turn modulate the gene expression in BOECs independent of physical contact, more studies are required to biochemically characterize these messengers/bioactive agents released from spermatozoa that eventually lead to the observed alterations in gene expression. It would undoubtedly be insightful to pursue the identification of their specific composition and target receptors on BOECs along with the unfolding signalling mechanisms which ultimately lead to the change in the transcriptomic profile of the oviductal epithelial cells. Based on the current knowledge of mediators of intercellular communication, these signals could be mediated by proteins or cell-free nucleic acids, including those carried by extracellular vesicles. Notably, it has been suggested that many proteins and exosomes are bound to spermatozoa surface (Kasvandik et al. 2015; Samanta et al. 2018), some of which could mediate the effects observed in this study. Biochemical characterisation of signals released by spermatozoa may eventually contribute to better diagnostics and treatment for male infertility problems of various aetiology.

While our study gives a general idea of temporal gene expression alterations, we note that we did not bridge the gap between 4-h responses (qPCR) and 10-h responses (RNAseq), the extent to which short-term responses differ from long-term responses remains unclear. Therefore, in future a long-term time-course analysis should be taken into consideration. There is abundant room for further progress in determining how specific these signals are, from spermatozoa to oviductal epithelial cells and if these signals would be the same for any other cell-type.

Finally, we must acknowledge two potential confounding factors. Firstly, although the percoll gradient is likely to have removed the majority of dead spermatozoa and we observed that the majority of spermatozoa were motile following the percoll wash, we cannot rule out that some oviductal cell responses could have been elicited by membrane components released by damaged sperm. Secondly, the responses may also have been influenced by composition of the cryo-protectant extender (commonly used in semen straw preparations), which has been shown previously to influence specific parameters of the spermatozoon including calcium fluxes, viability and acrosome morphology. However, such an effect would have been similar in spermatozoa in direct and non-direct contact with oviductal cells (Graham and Foote 1987; Zhao and Buhr 1995).

## Conclusion

Overall, the findings of our study shed new light on the communication between spermatozoa and the oviductal cells. Our results show for the first time that contact between spermatozoa and oviductal epithelial cells is not necessary to induce changes in the gene expression profile of the oviductal cells, suggesting that spermatozoa release signals that are capable of inducing alterations in gene expression profile of the recipient oviductal cells. Furthermore, we can deduce that the function of the spermatozoa is possibly not only confined to fertilization, but they may mediate other reproductive processes.

## Electronic supplementary material

ESM 1 (DOCX 28 kb)

## Data Availability

The sequencing data has been uploaded to the NCBI SRA repository under the BioProject ID: PRJNA624992.

## Abbreviations

BOECs: Bovine oviductal epithelial cells
EVs: Extracellular vesicles
DEGs: Differentially expressed genes
RNAseq: RNA sequencing
