# Supplementary material for: Spermatozoa induce transcriptomic alterations in bovine oviductal epithelial cells prior to initial contact
Source: J Cell Commun Signal. 2020 Sep 3;14(4):439–51. doi: 10.1007/s12079-020-00575-2 (PMC7642185; doi:10.1007/s12079-020-00575-2)
Supplement: Supplementary file 1 — (DOCX 28 kb) [file 12079_2020_575_MOESM1_ESM.docx]

**Table SI.** List of significantly differentially expressed genes (FDR<0.05) in control versus contact co-culture.

| **Gene symbol** | **log_2_FC** | **FDR** | **ENSEMBL ID** |
| --- | --- | --- | --- |
| *DHRS3* | 1.502 | 2.29e-42 | ENSBTAG00000024493 |
| *GPX1* | 0.667 | 1.43e-07 | ENSBTAG00000054195 |
| *PLAT* | 0.529 | 9.35e-05 | ENSBTAG00000001244 |
| *BHLHE40* | 0.491 | 0.0001 | ENSBTAG00000009863 |
| *ATF3* | 0.614 | 0.0001 | ENSBTAG00000008545 |
| *TNFRSF11B* | 0.721 | 0.0019 | ENSBTAG00000007423 |
| *SCIN* | 0.447 | 0.0035 | ENSBTAG00000009786 |
| *PLAU* | 0.413 | 0.0060 | ENSBTAG00000005947 |
| *CYP26A1* | 0.935 | 0.0060 | ENSBTAG00000021118 |
| *RANBP3* | -0.788 | 0.028 | ENSBTAG00000006070 |

**Table SII.** List of significantly differentially expressed genes (FDR<0.05) in control versus non-contact co-culture.

| **Gene Symbol** | **log_2_FC** | **FDR** | **Ensembl ID** |
| --- | --- | --- | --- |
| *CYP1A1* | 4.282 | 3.81e-123 | ENSBTAG00000001021 |
| *CYP1B1* | 2.173 | 1.49e-19 | ENSBTAG00000010531 |
| *TXNRD1* | 0.831 | 2.95e-16 | ENSBTAG00000013912 |
| *DHRS3* | 0.923 | 9.01e-15 | ENSBTAG00000024493 |
| *PTGS2* | 0.992 | 7.72e-10 | ENSBTAG00000014127 |
| *TIPARP* | 0.619 | 3.82e-09 | ENSBTAG00000012120 |
| *TNFRSF11B* | 0.923 | 1.31e-07 | ENSBTAG00000007423 |
| *ADGRF1* | 0.627 | 1.40e-06 | ENSBTAG00000021609 |
| *UGT1A6* | 1.862 | 7.66e-06 | ENSBTAG00000026181 |
| *UGT1A1* | 1.862 | 7.66e-06 | ENSBTAG00000026181 |
| *PLAU* | 0.491 | 4.05e-05 | ENSBTAG00000005947 |
| *BNIP3* | 0.888 | 4.05e-05 | ENSBTAG00000017804 |
| *PLA2G4A* | 0.491 | 0.0002 | ENSBTAG00000013298 |
| *PTGDR* | 0.544 | 0.0005 | ENSBTAG00000006703 |
| *RESF1* | 0.457 | 0.0005 | ENSBTAG00000004999 |
| *SLC2A1* | 0.475 | 0.0005 | ENSBTAG00000009617 |
| *MYH15* | 0.712 | 0.001 | ENSBTAG00000018399 |
| *RAI14* | 0.473 | 0.002 | ENSBTAG00000007071 |
| *GDF15* | 0.414 | 0.003 | ENSBTAG00000015618 |
| *TNFSF15* | 0.592 | 0.003 | ENSBTAG00000018069 |
| *MARCH3* | 0.456 | 0.004 | ENSBTAG00000006797 |
| *TBRG1* | 0.559 | 0.004 | ENSBTAG00000009410 |
| *N4BP2L1* | 0.469 | 0.005 | ENSBTAG00000000993 |
| *SERPINE2* | 0.399 | 0.006 | ENSBTAG00000008717 |
| *SCIN* | 0.399 | 0.006 | ENSBTAG00000009786 |
| *RND3* | 0.398 | 0.006 | ENSBTAG00000039731 |
| *DCBLD2* | 0.434 | 0.006 | ENSBTAG00000014889 |
| *SLC14A1* | 0.407 | 0.006 | ENSBTAG00000019870 |
| *ANGPTL4* | 0.631 | 0.007 | ENSBTAG00000002473 |
| *KDM3A* | 0.425 | 0.008 | ENSBTAG00000013580 |
| *CXCR4* | 0.379 | 0.013 | ENSBTAG00000001060 |
| *ISG20* | 1.069 | 0.013 | ENSBTAG00000014762 |
| *DSEL* | 0.523 | 0.014 | ENSBTAG00000037907 |
| *MICU1* | 0.362 | 0.015 | ENSBTAG00000005807 |
| *BIRC6* | 0.397 | 0.016 | ENSBTAG00000027932 |
| *AK4* | 0.543 | 0.016 | ENSBTAG00000030674 |
| *FUT6* | 0.512 | 0.016 | ENSBTAG00000000414 |
| *CEP350* | 0.400 | 0.016 | ENSBTAG00000008082 |
| *GPR35* | 0.789 | 0.020 | ENSBTAG00000030193 |
| *CFLAR* | 0.500 | 0.024 | ENSBTAG00000010998 |
| *MT1A* | 1.228 | 0.025 | ENSBTAG00000054808 |
| *DHODH* | 0.695 | 0.026 | ENSBTAG00000019887 |
| *CHAF1B* | 0.650 | 0.028 | ENSBTAG00000011880 |
| *SNX9* | 0.367 | 0.029 | ENSBTAG00000006323 |
| *MT2A* | 0.593 | 0.029 | ENSBTAG00000023659 |
| *ZNF317* | 0.446 | 0.030 | ENSBTAG00000016077 |
| *BRAP* | 0.484 | 0.031 | ENSBTAG00000001168 |
| *NFYB* | 0.425 | 0.031 | ENSBTAG00000030744 |
| *GRHPR* | 0.545 | 0.033 | ENSBTAG00000019299 |
| *LBR* | 0.429 | 0.034 | ENSBTAG00000008453 |
| *RPIA* | 0.392 | 0.038 | ENSBTAG00000002866 |
| *WDR45B* | 0.360 | 0.042 | ENSBTAG00000003695 |
| *CDKN2D* | 0.713 | 0.043 | ENSBTAG00000010731 |
| *CLDN1* | -0.715 | 1.64e-10 | ENSBTAG00000013148 |
| *PRSS23* | -0.635 | 3.48e-06 | ENSBTAG00000015177 |
| *CD151* | -0.673 | 5.17e-06 | ENSBTAG00000019569 |
| *PTK7* | -0.928 | 3.59e-05 | ENSBTAG00000012761 |
| *BCAM* | -0.615 | 0.0001 | ENSBTAG00000009495 |
| *VAT1* | -0.620 | 0.0004 | ENSBTAG00000007390 |
| *GALK1* | -0.750 | 0.0023 | ENSBTAG00000014964 |
| *IKBIP* | -0.902 | 0.0023 | ENSBTAG00000021660 |
| *IFRD2* | -0.646 | 0.003 | ENSBTAG00000000480 |
| *ZFYVE21* | -0.979 | 0.003 | ENSBTAG00000003556 |
| *P2RY2* | -0.715 | 0.003 | ENSBTAG00000039050 |
| *NA* | -0.74 | 0.003 | ENSBTAG00000048781 |
| *RPS9* | -0.55 | 0.004 | ENSBTAG00000006487 |
| *TNC* | -0.46 | 0.004 | ENSBTAG00000000575 |
| *SPSB3* | -0.91 | 0.006 | ENSBTAG00000016558 |
| *ACIN1* | -0.79 | 0.006 | ENSBTAG00000011571 |
| *TMC6* | -0.63 | 0.011 | ENSBTAG00000018661 |
| *SBSPON* | -0.69 | 0.013 | ENSBTAG00000017249 |
| *BCKDHB* | -0.51 | 0.013 | ENSBTAG00000012096 |
| *ATG3* | -0.38 | 0.014 | ENSBTAG00000009084 |
| *EHD2* | -0.68 | 0.014 | ENSBTAG00000021191 |
| *EIF3F* | -0.49 | 0.015 | ENSBTAG00000004861 |
| *DDX54* | -0.76 | 0.016 | ENSBTAG00000011930 |
| *EFEMP1* | -0.34 | 0.016 | ENSBTAG00000017448 |
| *CCND1* | -1.02 | 0.016 | ENSBTAG00000017514 |
| *POLR2L* | -0.47 | 0.016 | ENSBTAG00000031061 |
| *NPDC1* | -1.23 | 0.016 | ENSBTAG00000046140 |
| *QSOX1* | -0.45 | 0.016 | ENSBTAG00000014191 |
| *RPS2* | -0.38 | 0.017 | ENSBTAG00000009535 |
| *S100A10* | -0.38 | 0.021 | ENSBTAG00000015147 |
| *PTGDS* | -0.56 | 0.022 | ENSBTAG00000015074 |
| *CCNA2* | -0.64 | 0.022 | ENSBTAG00000004943 |
| *MAOA* | -0.85 | 0.022 | ENSBTAG00000016206 |
| *COL4A3BP* | -0.68 | 0.022 | ENSBTAG00000000081 |
| *SLC35F5* | -0.41 | 0.022 | ENSBTAG00000021103 |
| *SDC4* | -0.51 | 0.024 | ENSBTAG00000015127 |
| *TM4SF1* | -0.52 | 0.027 | ENSBTAG00000015163 |
| *CREB3L2* | -0.70 | 0.027 | ENSBTAG00000015802 |
| *ADAMTS15* | -0.92 | 0.027 | ENSBTAG00000016857 |
| *DEDD2* | -0.63 | 0.028 | ENSBTAG00000005761 |
| *DTWD2* | -1.48 | 0.030 | ENSBTAG00000002143 |
| *CDKN2A* | -0.73 | 0.031 | ENSBTAG00000034220 |
| *LMNB1* | -0.77 | 0.033 | ENSBTAG00000002882 |
| *GCN1* | -0.54 | 0.035 | ENSBTAG00000017379 |
| *GPAT4* | -0.97 | 0.036 | ENSBTAG00000005730 |
| *MYL9* | -0.56 | 0.036 | ENSBTAG00000011473 |
| *INHBA* | -0.47 | 0.036 | ENSBTAG00000048508 |
| *EHD1* | -0.40 | 0.038 | ENSBTAG00000050712 |
| *STIM1* | -0.44 | 0.040 | ENSBTAG00000013109 |
| *NA* | -0.95 | 0.040 | ENSBTAG00000046100 |
| *SART3* | -0.55 | 0.040 | ENSBTAG00000031846 |
| *SERPINB10* | -0.44 | 0.041 | ENSBTAG00000001102 |
| *CDC42EP3* | -0.34 | 0.042 | ENSBTAG00000023736 |
| *MCUB* | -0.64 | 0.043 | ENSBTAG00000012995 |
| *TMEM37* | -0.50 | 0.044 | ENSBTAG00000026825 |
| *CAPN2* | -0.33 | 0.048 | ENSBTAG00000012778 |
